# Human papillomavirus (HPV) in Chinese oropharyngeal squamous cell carcinoma (OPSCC): A strong predilection for the tonsil

**DOI:** 10.1002/cam4.3339

**Published:** 2020-07-27

**Authors:** Tingting Xu, Chunying Shen, Ye Wei, Chaosu Hu, Yu Wang, Jun Xiang, Guo‐Hua Sun, Fengtao Su, Qifeng Wang, Xueguan Lu

**Affiliations:** ^1^ Department of Radiation Oncology Fudan University Shanghai Cancer Center Shanghai China; ^2^ Department of Oncology Shanghai Medical College of Fudan University Shanghai China; ^3^ Department of Head and Neck Surgery Fudan University Shanghai Cancer Center Shanghai China; ^4^ Cancer Institute Fudan University Shanghai Cancer Center Shanghai China; ^5^ Department of Pathology Fudan University Shanghai Cancer Center Shanghai China

**Keywords:** EBV, HPV, oropharyngeal squamous cell carcinoma, prognosis, tonsillar

## Abstract

**Objective:**

Compared with Occident's data, the incidence of Human papillomavirus (HPV)‐driven oropharyngeal squamous cell carcinomas (OPSCCs) had been reported as relatively low in Mainland China. The objective of this study was to report the integrated prevalence of HPV and Epstein‐Barr virus (EBV), and further evaluate the different behaviors of HPV‐positive and ‐negative OPSCCs in eastern China.

**Methods:**

In a cohort of 170 nonmetastatic OPSCCs treated from January 2007 to July 2019, p16 protein expression, HPV genotypes, and Epstein‐Barr virus‐encoded RNA (EBER) were determined by immunohistochemistry (IHC) and in situ hybridization (ISH). The clinical and pathologic findings were further collected and analyzed to comprehensively reveal the behaviors of Chinese OPSCCs.

**Results:**

Out of the 170 tumor tissues evaluated, 57.6% (98) samples had positive p16 expressions. A total of 65.1% (99/152) samples had positive HPV genotypes, besides HPV16 (92/152), HPV11, 18, 33, 53, and 58 were also detected. The positive rate of EBER was 7.2% (9/124), and the co‐infection rate of EBV/HPV was 4.0%. Related to the unequal distributions of p16 expression, HPV‐related tumors arisen from tonsillar and non‐tonsillar accounted for 68.8% (75/109) and 37.7% (23/61) of their cases, respectively (*P* < .001). With a median follow‐up time of 13.1 months, significant survival advantages of HPV‐related OSPCC were observed; 1‐year OS, PFS, RFS, and MFS were 83.2% vs 96.7% (*P* < .001), 71.6% vs 96.2% (*P* < .001), 77.7% vs 96.2% (*P* = .002), and 90.4% vs 100.0% (*P* = .024) in p16‐negative and ‐positive cases, respectively.

**Conclusions:**

The relative percent of HPV‐positive OPSCCs in this study is close to the positive rate in many Western countries and a strong predilection was discovered for the tonsillar. The EBV infection and co‐infection of HPV/EBV were largely low. The prognosis of HPV‐positive OSPCCs was more favorable than its negative counterpart.

## Graphical abstract

HPV‐positive OPSCC in China resembled with Western countries. EBV infection and co‐infection of HPV/EBV were low in OPSCC. The prognosis of HPV‐positive OSPCC was more favorable.

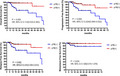

## 1 INTRODUCTION

Referring to the clinical therapeutic strategies of OPSCCs, HPV is the key determinate factor both for treatment efficacy and long‐term survival prediction.^1^, ^2^, ^3^, ^4^ Disparities of treatment responses have been observed irrespective of clinical stage between different HPV statuses. Furthermore, there was considerable discrepancy of HPV infection rates between eastern and western countries due to different geographical settings and the different lifestyles including sexual cultures, smoking, and drinking habits.^5^, ^6^ In the past few years, some authors reported the HPV infection of oropharyngeal carcinoma in China and found largely low incidence of both p16 and HPV subtypes in their institutions,^7^, ^8^, ^9^ which was 11%‐15% compared with approximately 50%‐70% in a large part of western countries.^4^, ^10^, ^11^ Nevertheless, due attention should be given to the primary location as oropharyngeal carcinoma arisen from various parts (tonsil, soft palate, base of tongue, and posterior wall) may have distinct features and etiologic factors. Furthermore, the Epstein‐Barr virus (EBV) infection and co‐infection of HPV/EBV were never reported in Chinese patients.

We hereby evaluated 170 OPSCC tissues in eastern China with p16 protein expression, HPV genotypes and Epstein‐Barr virus‐encoded RNA (EBER). The clinical and pathologic findings were further collected and analyzed to comprehensively reveal the behaviors of tonsillar and non‐tonsillar squamous cell carcinoma.

## 2 MATERIAL AND METHODS

### 2.1 Patients and specimens collection

A total of 170 nonmetastatic squamous carcinoma of oropharynx patients treated in our Centre from January 2007 to July 2019 with accessible p16 IHC results were reviewed, and those (158 cases) with integrated age, sex, smoking history, TNM stage, treatment regimen, and follow‐up information were enrolled in survival analyses. The study was approved by the local Ethics Committee and was conducted in accordance with the ethical principles. All the MRI imaging slices were reviewed and the clinical tumor (T), nodal (N), and distant metastasis (M) categories were re‐defined using the American Joint Commission on Cancer 8th edition guidelines.

Once diagnoses were confirmed, patients were thoroughly treated after multidisciplinary team (MDT) discussion. Treatment modalities included surgery followed by postoperative radiotherapy/chemoradiotherapy and radical radiotherapy (intensity‐modulated radiation therapy, IMRT) with/without cisplatin‐based chemotherapy. In brief, the prescription doses of postoperative radiotherapy were 60 Gy/30 Fx to tumor bed + high‐risk drainage area and 54Gy/30Fx to the low‐risk drainage area. The radical doses were 60‐70 Gy/30‐35 Fx, 60‐63 Gy/30‐35 Fx, and 54‐56 Gy/30‐35 Fx to gross‐tumor volume (GTV), high‐risk clinical tumor volume CTV (CTV1), and low‐risk CTV (CTV2), respectively.

### 2.2 IHC of p16, ISH of HPV genotypes and EBER

Commercial antibody of p16 was used to detect the p16 expression of all 170 patients, using p16 immunohistochemistry with a 70% nuclear and cytoplasmic staining cutoff. IHC was performed on 4‐µm sections of paraffin‐embedded tissues to determine the expression level of p16 protein. In brief, the slides were incubated in p16 antibody (M78710, Dako) diluted 1:200 at 4°C overnight and incubated in second antibody (Dako) at 37°C for 40 minutes. Then the slides were stained with the avidin–biotin‐peroxidase method with DAB (diaminobenzidine) and counterstained with hematoxylin. Three times washing with PBS were done before and after steps. The specific steps were performed using the EnVision™ FLEX High pH visualization system (Dako) according to the manufacturer's instructions. Slides were examined under light microscope for evaluation.

In 152 specimens that yielded valid results, ISH study was used to test OPSCC patients harboring 18 high‐risk and 5 low‐risk HPV genotypes. The specific steps were performed using the Wide Spectrum HPV Test Kit (Triplex International Biosciences) with signal magnifying system according to the manufacturer's instructions. A quantity of 25 µL wide spectrum HPV probe was added to the tissue section and the slides were incubated at 37°C for 4‐16 hours in a humidity chamber with 30% formamide solution. The liquid was tapped off and wiped around the tissue section; a drop (about 50 µL) of mouse anti‐Dig antibody was added and incubated at 37°C in a humidity chamber with distilled water for 30 minutes. A drop (50 µL) of polymerized HRP‐anti mouse IgG was added and incubated at room temperature in a humidity chamber with distilled water for 30 minutes. A drop (50 µL) of DAB solution was prepared immediately before use. Three washings with PBS were done before and after steps. The results were interpreted under the microscope. A total of 18 high‐risk (HPV16, 18, 31, 33, 35, 39, 45, 51, 52, 53, 56, 58, 59, 66, 68, 73, 83, 82) and 5 low‐risk HPV subtypes (HPV6, 11, 42, 43, 81) were tested.

EBER ISH study was performed on 4 µm thick formalin‐fixed paraffin‐embedded sections. The specific steps were performed using the EBER Test Kit (Triplex International Biosciences) with signal magnifying system according to the manufacturer's instructions. After proteinase K digestion, 25 µL of EBER Probe was dropped into the tissue section and the slides were incubated at 55°C for 60‐90 minutes and at 37°C for 4‐16 hours in a humidity chamber. Mouse anti‐Dig Antibody, polymer enhancer, and polymerized HRP‐Anti Mouse IgG, were used for signal magnifying. DAB solution was used for signal display. Three washings with PBS were done before and after steps. The results were interpreted under the microscope.

All the tests were judged by two independent pathologists.

### 2.3 Endpoints and statistics

Assessments of the response rates were performed by the physicians using the RECIST (Response Evaluation Criteria in Solid Tumors) v1.1 criterion. Statistical tests were analyzed using SPSS 20.0 software (SPSS, Inc). Comparisons of clinical and general features between the p16 and HPV genotypes level groups were conducted using Pearson chi‐square test or Fisher's exact test. Overall survival (OS) was defined as the time from the beginning of treatment to death as a result of any cause. Relapse‐free survival (RFS) and metastasis‐free survival (MFS) were defined as the duration from the date of treatment to relapse or distant metastasis. In the analysis of progression‐free survival (PFS), a patient was considered to have progressed if he relapsed/metastasized after the completion of all primary treatment. Survival analyses were assessed using the Kaplan‐Meier method and log‐rank (Mantel‐Cox) test. A value of *P* < .05 was considered to be statistically significant.

## 3 RESULTS

### 3.1 Expression of p16, HPV genotypes, and EBER in OPSCC tumor samples

Out of the 170 tumor tissues evaluated, 42.4% (72) samples had negative and 57.6% (98) had positive p16 expressions. Of the 152 HPV genotype‐evaluable patients, a total of 65.1% (99) samples had positive staining, most of them (92 samples, 60.5%) were HPV16; besides, HPV11 (0.7%), HPV18 (0.7%), HPV33 (0.7%), HPV53 (0.7%), and HPV58 (2.0%) were also detected. Nonuniform distribution of both p16 and HPV genotypes were discovered in tumors arising from different regions of the oropharynx, a significant divergence was observed in tonsillar and non‐tonsillar carcinoma (Table 1). According to the p16 status, HPV‐related tonsillar and non‐tonsillar SCC accounted for 68.8% (75/109) and 37.7% (23/61) of the diseases arisen from the corresponding locations, respectively (*P* < .001).

**TABLE 1:** Nonuniform distribution of p16 and HPV genotypes in different regions of the oropharynx

Location	Stratified by p16 IHC (n = 170)			Stratified by HPV genotypes (n = 152)		
	p16− (%)	p16+ (%)	*P*	HPV− (%)	HPV+ (%)	*P*
Tonsil	34 (20.0)	75 (44.1)	.000	25 (16.4)	70 (46.1)	.004
Base of tongue	25 (14.7)	18 (10.6)		17 (11.2)	23 (15.1)	
Soft palate	10 (5.9)	5 (2.9)		8 (5.3)	6 (3.9)	
Posterior wall	3 (1.8)	0 (0)		3 (2.0)	0 (0)	

Among 124 patients with accessible EBER results, a total of 7.2% (9) samples had positive staining and 5 (4.0%) were defined EBV/HPV co‐infection (4 of them were HPV16).

### 3.2 Patient and disease characteristics and relationships with different p16/HPV genotypes

Baseline characteristics of the 170‐patient study population classified by p16/HPV genotype subgroups were broadly variant; obviously, patients with p16‐positive OPSCC had lower tumor and nodal stages, had lower rates of heavy/ever‐smoking and alcohol consumption, and more younger females. Notwithstanding, the T stage and the characteristics of involved neck and retropharyngeal lymph nodes were generally similar (Table 2). Totally, there were 154 patients treated in our center after confirmed diagnosis and complete inspection of the disease, including 110 radiotherapy alone or chemoradiotherapy and 44 surgeries followed by RT/CRT. In the subgroup analyses, correlations were significant between p16 expression and short‐term response rates (RR) in 110 patients received nonsurgical treatment (including radiotherapy alone or chemoradiotherapy) (Table 3). In addition, we observed significantly higher risks of post‐treatment mucosal ulcer (PTMU) in p16‐negative group, which were totally 8 out of 170 stand for a risk of 4.7%. The criteria for diagnosing PTMU at MRI imaging were discontinuous oropharyngeal mucosa line and/or a focal area of low signal intensity on contrast‐enhanced T1‐weighted images. Furthermore, the secondary primary tumor were also more common in p16‐negative patients, hereby further clarified the reasons for its unfavorable prognosis (Table 4).

**TABLE 2:** Baseline patients' characteristics stratified by p16 and HPV genotypes

	Stratified by p16 IHC (n = 170)			Stratified by HPV genotypes (n = 152)		
	p16− (%)	p16+ (%)	*P*	HPV− (%)	HPV+ (%)	*P*
Sex			.000			.030
Male	68 (40.0)	72 (42.4)		49 (32.2)	78 (51.3)	
Female	4 (2.3)	26 (15.3)		4 (2.6)	21 (13.8)	
Age (y)			.049			.046
Median (Range)	58.5 (36‐80)	55.5 (22‐76)		57 (36‐71)	56 (22‐80)	
Smoking			.000			.004
Never	19 (11.2)	58 (34.1)		17 (11.2)	52 (34.2)	
≤10PK·Y	2 (1.2)	9 (5.3)		1 (0.6)	8 (5.3)	
>10 PK·Y	51 (30)	31 (18.2)		35 (23.0)	39 (25.7)	
Alcohol			.000			.019
Never	29 (17.1)	72 (42.3)		25 (16.4)	66 (43.4)	
Yes	43 (25.3)	26 (15.3)		28 (18.4)	33 (21.7)	
T stage			.183			.051
T1	15 (8.8)	20 (11.8)		9 (5.9)	22 (14.5)	
T2	24 (14.1)	46 (27.1)		19 (12.5)	44 (28.9)	
T3	22 (12.9)	25 (14.7)		14 (9.2)	27 (17.8)	
T4	11 (6.5)	7 (4.1)		11 (7.2)	6 (3.9)	
N stage			.000			.000
N0	7 (4.1)	6 (3.5)		2 (1.3)	6 (3.9)	
N1	7 (4.1)	54 (31.8)		6 (3.9)	55 (36.2)	
N2	9 (5.3)	29 (17.1)		9 (5.9)	31 (20.5)	
N3a	1 (0.6)	/		1 (0.6)	/	
N3b	48 (28.2)	/		35 (23.0)	/	
N3	/	9 (5.3)		/	7 (4.6)	
TNM stage			.000			.000
I	6 (3.5)	40 (23.5)		2 (1.3)	42 (27.6)	
II	2 (1.2)	43 (25.3)		3 (2.0)	44 (28.9)	
III	6 (3.5)	15 (8.8)		6 (3.9)	13 (8.6)	
IVa	9 (5.3)	/		6 (3.9)	/	
IVb	49 (28.9)	/		36 (23.7)	/	
Max size of LN (cm)			.645			.187
0	7 (4.1)	6 (3.5)		2 (1.3)	6 (3.9)	
≤3	28 (16.5)	42 (24.8)		26 (17.1)	39 (25.7)	
3‐6	30 (17.6)	44 (25.9)		18 (11.8)	48 (31.6)	
>6	7 (4.1)	6 (3.5)		7 (4.6)	6 (3.9)	
LN locations			.299			.599
None	7 (4.1)	6 (3.5)		2 (1.3)	6 (3.9)	
Ipsilateral	38 (22.4)	63 (37.1)		30 (19.7)	61 (40.1)	
Bilateral	27 (15.9)	29 (17.0)		21 (13.8)	32 (21.1)	
ENE			.787			.755
No	25 (14.7)	36 (21.2)		19 (12.5)	33 (21.7)	
Yes	47 (27.6)	62 (36.5)		34 (22.4)	66 (43.4)	
RLN			.498			.315
None	43 (25.3)	60 (35.4)		29 (19.1)	63 (41.5)	
Ipsilateral	22 (12.9)	33 (19.4)		18 (11.8)	31 (20.4)	
Bilateral	7 (4.1)	5 (2.9)		6 (3.9)	5 (3.3)	
Treatment			.025			.000
RT/CRT	49 (31.8)	61 (39.6)		40 (29.4)	57 (41.9)	
S ± RT/CRT	11 (7.1)	33 (21.4)		4 (2.9)	35 (25.7)	

Abbreviations: CRT, chemoradiotherapy; ENE, extranodal extension; LN, lymph node; PK·Y, pack·year; RLN, retropharyngeal lymph node; RT, radiotherapy; S, surgery.

**TABLE 3:** Correlations between p16 IHC and treatment response rates (RR) for primary tumor and lymph nodes (n = 110)

PT	p16− n (%)	p16+ n (%)	*P*	LN	p16− n (%)	p16+ n (%)	*P*
CR	33 (30.0)	57 (51.8)	.002	CR	21 (19.1)	48 (43.6)	.001
PR	13 (11.8)	4 (3.6)		PR	21 (19.1)	12 (10.9)	
SD	2 (5.9)	0 (0)		SD	5 (4.5)	1 (0.9)	
PD	1 (1.8)	0 (0)		PD	2 (1.8)	0 (0)	

Abbreviations: CR, complete response; LN, Lymph node; PD, progression disease; PR, partial response; PT, primary tumor; SD, stable disease.

**TABLE 4:** Risk of occurrence of post‐treatment oropharyngeal mucosal ulcer and secondary primary tumor (n = 170)

	p16‐ (%) (n = 72)	p16+ (%) (n = 98)	*P*
Mucosal ulcer			.011
No	65 (38.2)	97 (57.1)	
Yes	7 (4.1)	1 (0.6)	
Secondary primary tumor			.043
No	66 (38.8)	97 (57.1)	
Yes	6 (3.5)	1 (0.6)	

### 3.3 Impact of p16/HPV genotypes and predictive role of smoking on survival

**FIGURE 1:**
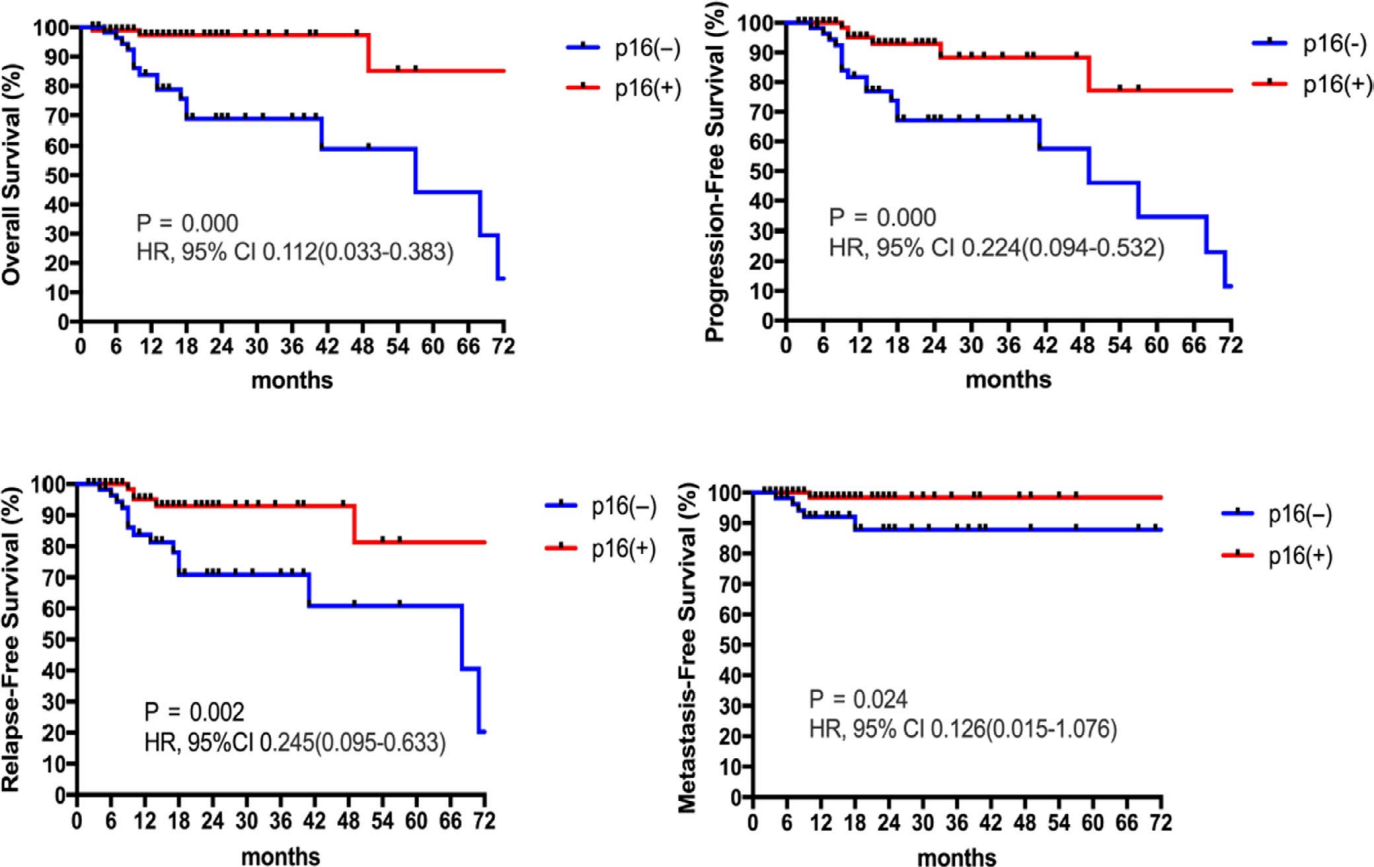
Survival curves stratified by p16 IHC status showed favorable outcomes in positive populations

Survival analyses were conducted in 154 patients with integrated treatment regimen and follow‐up information. At the time of analysis for survival (February 9, 2020), 20 OS, 26 PFS, 21 RFS, and 6 MFS events had occurred with a median follow‐up time of 13.1 months (1.7‐99.1 months). There were significant survival advantages of HPV‐related OSPCCs according to p16 protein expressions, as showed in Figure 1. We further calculated the prognosis stratified by HPV genotype status (44 negative and 92 positive) and disease locations (102 tonsillar and 52 non‐tonsillar) (Table 5). HPV positive is associated with improved 1‐year OS and RFS and tonsillar squamous cell carcinoma is associated with improved 1‐year PFS.

**TABLE 5:** Survival rates of OPSCC patients with different p16/HPV status and primary tumor locations

	p16− (%) (n = 72)	p16+ (%) (n = 98)	*P*	HR, 95% CI
1‐year OS	83.2	96.7	<.001	0.112 (0.033‐0.383)
1‐year PFS	71.6	96.2	<.001	0.224 (0.094‐0.532)
1‐year RFS	77.7	96.2	.002	0.245 (0.095‐0.633)
1‐year MFS	90.4	100	.024	0.126 (0.015‐1.076)

	HPV− (%) (n = 44)	HPV+ (%) (n = 92)	*P*	HR, 95% CI
1‐year OS	83.0	94.1	.037	0.402 (0.166‐0.973)
1‐year PFS	77.3	88.5	.106	0.526 (0.238‐1.164)
1‐year RFS	79.2	91.5	.037	0.403 (0.167‐0.974)
1‐year MFS	97.6	95.2	.473	2.156 (0.251‐18.507)

	Non‐tonsil (%) (n = 52)	Tonsil (%) (n = 102)	*P*	HR, 95% CI
1‐year OS	89.7	93.3	.128	0.504 (0.205‐1.238)
1‐year PFS	79.7	90.8	.046	0.453 (0.204‐1.005)
1‐year RFS	84.3	91.9	.068	0.446 (0.184‐1.084)
1‐year MFS	95.3	96.9	.763	0.780 (0.155‐3.927)

The Cox proportional hazard model included p16 status (negative vs positive), sex (male vs female), age (≤58 years vs >58 years), smoking (never vs ever), alcohol consumption (no vs yes), tonsillar (no vs yes), treatment regimen (CRT vs surgery ± RT/CRT), and post‐treatment mucosal ulcer (no vs yes) (Table 6). In multivariate analysis, OS and PFS were only independently affected by the presence of p16 status and post‐treatment mucosal ulcer. However, other factors were not proved to do harmful influence on survival due to the short median follow‐up time.

**TABLE 6:** Multivariate analysis of prognostic factors

	1y OS (%)		*P*	HR, 95% CI	1y PFS (%)		*P*	HR, 95% CI
P16	83.2	96.7	.008	0.142 (0.034‐0.600)	71.6	96.2	.032	0.336 (0.124‐0.912)
Sex	89.3	100	.862	0.823 (0.092‐7.375)	83.3	84.5	.234	0.281 (0.035‐2.278)
Age	89.9	92.7	.757	1.185 (0.404‐3.478)	88.0	84.5	.906	1.053 (0.449‐2.468)
Smoking	95.2	86.9	.790	0.834 (0.219‐3.177)	93.6	78.8	.384	0.627 (0.219‐1.794)
Alcohol	93.0	88.1	.477	1.541 (0.468‐5.081)	92.4	76.0	.220	1.905 (0.681‐5.332)
Tonsillr	93.3	89.7	.937	1.040 (0.396‐2.729)	79.7	90.8	.684	0.835 (0.351‐1.989)
Treatment	92.6	88.2	.091	2.455 (0.866‐6.966)	86.3	86.3	.458	1.408 (0.571‐3.472)
Mucosal ulcer	94.5	28.6	.001	8.162 (2.392‐27.854)	90.1	23.4	.007	4.882 (1.531‐15.571)

## 4 DISCUSSION

During the past decade, a series of studies had noted the HPV as determining factor of OPSCC for prognosis.^1^, ^2^, ^3^, ^4^ HPV‐related OPSCCs represent good responses to treatment and long‐term survival, which seems the intensity of management should be decreased to reduce toxicities.^12^, ^13^, ^14^ Therefore, the appropriate population and optimal strategy for treatment de‐escalation is under active investigation. On the other hand, we had been well informed about the distinction of pathology and biological behavior of nasopharyngeal carcinoma (NPC) between epidemic areas (especially southern China) and Western countries. As an extension of nasopharynx with no natural barrier to each other, together with the similar etiology (caused by virus), can we simply apply the results of Western studies to Chinese patients? However, few studies can be obtained to get an unequivocal conclusion.

The Chinese mainland‐based results of OPSCC in this study confirmed the discovery of Occident's reports in the past decade. As reported, patients with HPV‐positive OPSCC tended to be younger, less exposure to tobacco/alcohol, with smaller primary tumors, but early nodal metastases diseases.^15^, ^16^ Similar differences of baseline characteristics were founded in the current retrospective analyses, HPV‐related patients had lower tumor and nodal stages (owing to the different staging criterion), had lower rates of heavy/ever‐smoking and alcohol consumption, and more younger females compared with their HPV‐negative counterparts, which means analogous biological behavior to previous studies.

In the past few years, several studies from Mainland China reported the incidence of HPV‐related OPSCC (approximately 5%‐15%) as well as unhealthy lifestyle (mainly smoking and alcohol) induced carcinoma; however, they were unable to well distinguish the HPV infection rates among different anatomic lesions.^7^, ^8^, ^9^ In Xu et al's recent study,^9^ the overall HPV infection rate was found to be 18.29%, whereas only 18 out of 257 their OPSCC were tonsil carcinomas, and 44.4% (8/18) were both p16 IHC and HPV ISH positive. They merely described a trend of higher infection rate due to the relatively small sample size of tonsil carcinoma patients. Besides, some research had pointed out that the reticulated tonsillar crypt epithelium of the oropharynx, characteristic of the palatine tonsils and lingual tonsillar subsites, appeared to be especially susceptible to HPV infection and subsequent malignant transformation.^17^ Also, whether the common phenomenon existed in Chinese patients is not clear. So more detailed distribution patterns should be addressed in order to precisely select the groups who would have better response to treatment and then we are able to set them apart from conventional chemoradiotherapy and deliver alternative therapeutic regimens with appropriate toxicities and complications, exactly called, the de‐escalation strategy. In our analyses, HPV‐related OPSCC had a strong predilection for the tonsillar (68.8%) but not the non‐tonsillar (37.7%) lesions. To our knowledge, this was the first report in Chinese population to explicit the distinct histogenesis and pathogenic factors of them precisely. As a result, the prognosis likewise showed differences. Tonsillar patients achieved significantly better PFS and marginally less local‐regional recurrence when compared with their non‐tonsillar companions.

According to the assessment from the Institut Català d'Oncologia (ICO) International HPV in Head and Neck Cancer Study Group, HPV16 contributed to the majority (83%) of HPV + HNC cases worldwide, meanwhile, HPV18 (1.8%), 19 (0.4%), 26 (2.6%), 30 (0.4%), 33 (3.3%), 35 (2.2%), 39 (0.4%), 45 (0.4%), 51 (0.7%), 53 (0.4%), 58 (0.7%), 59 (0.4%), 66 (0.4%), 68 (0.4%), and 69 (0.7%) were also detected.^18^ Most of their patients were from Europe and central‐South America, while our results expounded the similar distribution of HPV genotypes in Chinese populations (HPV16 accounted for 92.9% of all genotypes, HPV11, 18, 33, 53, and 58 were also detected).

Paradoxically, the two key factors to test the HPV infection status, p16 IHC and HPV RNA/DNA detection has been reported to be associated with a proportion of false positive and negative results especially in low‐HPV‐incidence regions, as almost 15%‐20% p16 IHC–positive cases are HPV negative.^19^, ^20^ Based on the ASCO Clinical Practice Guideline for HPV testing in head and neck carcinomas released in 2018 by the CAP (College of American Pathologists), both p16 and high‐risk (HR)‐HPV testing were recommended for newly diagnosis OPSCC.^21^ They mentioned that a small fraction of oropharyngeal tumors are not etiologically driven by HPV yet overexpress p16. Vice versa, a subset of patients with HPV‐negative can overexpress p16 (eg, as a result of a mutation in RB1). We considered looking for the potentially appropriate surrogate for HPV‐attributable OPSCC is of paramount importance. Actually, partial disaccords remained observed in our cohort. We revealed p16 IHC to be a more sensible method. Compared with ISH test, IHC negative will be better to signify an ominous prognosis.

The primary detection of cervical lymph nodes from an unknown primary site (SCCUP) represented approximately 5% of all head and neck squamous carcinomas (HNSCCs). Identification of the primary site is of paramount importance as it helps pertinent therapy to achieve better prognosis. The CAP has called for mandatory testing of all OPSCCs, either from oropharyngeal primary or metastatic lymph nodes, including lymph nodes of unknown primary origin.^21^ Bussu et al^22^ analyzed the HPV and EBV infections in neck nodes from occult primary SCC, more than half of nodes were positive for at least one virus, but co‐infection were only detected in three cases. Deng et al^23^ also found rare co‐infection (1.0%) in HNSCCs. In Broccolo et al's study,^24^ co‐infection was found only in 4 (10%) OPSCC and that were all in consistent with our calculation (only 7.2% OPSCC had EBV infection and 4.0% were co‐infection), which means p16/HPV genotype positive may highly hint the primary location for oropharynx. In the meantime, EBER‐positive SCCUP highly imply nasopharynx but is on the off chance of oropharynx. Occult tonsil and base of tongue lesions that are radiologically and endoscopically undetectable with confirmed metastatic neck nodes could be further diagnosed by biopsies of tonsil or base of tongue or even tonsillectomies.^25^

Given the prior evidence and potential mechanisms described, it may not be surprising that our study demonstrated the poor response to RT/CRT, local and distant disease control of HPV‐negative cases. What is worse, higher risks of post‐treatment oropharyngeal mucosal ulcer and secondary primary tumor were more common in p16‐negative patients, leading to unfavorable overall survival in those populations.

In conclusion, the present study demonstrated the positive proportions, biological behavior and characteristics of HPV‐related OPSCC were generally in accordance with previous findings in epidemic countries, and it had a strong predilection for the tonsillar. The treatment response rate and prognosis of HPV‐positive OSPCCs were more favorable than negative ones. Owing to the short median follow‐up time, we were not able to clarify the exact influence of disease characteristics on survival with the exception of p16 IHC and post‐treatment mucosal ulcer in multivariate analyses. With careful patients' selection, we can design our prospective protocols to achieve less toxicities and improve quality of life without compromising efficacies with the help of less intensive treatment regimens.

## CONFLICT OF INTEREST

The author(s) declared no potential conflicts of interest with respect to the research, authorship, and/or publication of this article.

## AUTHOR CONTRIBUTION

T. Xu and C. Shen: Statistical analysis and manuscript preparation/editing. Y. Wei: Manuscript preparation. C. Hu: Study design. Y. Wang, J. Xiang and G. Sun: Data acquisition. F. Su: Manuscript review. Q. Wang: Pathological procedure and diagnosis. X. Lu: Study concepts.

## Data Availability

The data that support the findings of this study are available from the corresponding author upon reasonable request.
